# Aberrant Otx2 Expression Enhances Migration and Induces Ectopic Proliferation of Hindbrain Neuronal Progenitor Cells

**DOI:** 10.1371/journal.pone.0036211

**Published:** 2012-04-27

**Authors:** Matthew Wortham, Genglin Jin, Julia Lailai Sun, Darell D. Bigner, Yiping He, Hai Yan

**Affiliations:** Department of Pathology, The Pediatric Brain Tumor Foundation Institute, and The Preston Robert Tisch Brain Tumor Center at Duke, Duke University Medical Center, Durham, North Carolina, United States of America; University of Florida, United States of America

## Abstract

Dysregulation of Otx2 is a hallmark of the pediatric brain tumor medulloblastoma, yet its functional significance in the establishment of these tumors is unknown. Here we have sought to determine the functional consequences of Otx2 overexpression in the mouse hindbrain to characterize its potential role in medulloblastoma tumorigenesis and identify the cell types responsive to this lineage-specific oncogene. Expression of Otx2 broadly in the mouse hindbrain resulted in the accumulation of proliferative clusters of cells in the cerebellar white matter and dorsal brainstem of postnatal mice. We found that brainstem ectopia were derived from neuronal progenitors of the rhombic lip and that cerebellar ectopia were derived from granule neuron precursors (GNPs) that had migrated inwards from the external granule layer (EGL). These hyperplasias exhibited various characteristics of medulloblastoma precursor cells identified in animal models of Shh or Wnt group tumors, including aberrant localization and altered spatiotemporal control of proliferation. However, ectopia induced by Otx2 differentiated and dispersed as the animals reached adulthood, indicating that factors restricting proliferative lifespan were a limiting factor to full transformation of these cells. These studies implicate a role for Otx2 in altering the dynamics of neuronal progenitor cell proliferation.

## Introduction

Medulloblastoma is an aggressive pediatric brain tumor whose treatment results in substantial long-term side effects [1]. An advanced understanding of the mutations driving medulloblastoma tumorigenesis has the potential to improve upon existing therapeutic strategies. Exome sequencing [2] and genomic copy number analysis [3] have revealed the common genomic alterations giving rise to medulloblastoma. However, despite an emerging understanding of the genetic underpinnings of this tumor, the functional consequences of many of these aberrations remain unclear.

Animal modeling has revealed key context-dependent functions of various medulloblastoma oncogenes and tumor suppressors. Many of the genomic aberrations present in medulloblastoma are molecular subtype-specific [4], [5], and the critical pathways affected have been shown to exert variable effects upon different cell types of the mouse hindbrain [6], [7]. Studies of the medulloblastoma-susceptible *Patched* ^+/−^ mouse and variations thereof have identified the critical mitogenic effect of Shh upon cerebellar GNPs and revealed this cell type to be the cell of origin for Shh group medulloblastomas [6], [8]. Similarly, modeling Wnt pathway activation in the mouse hindbrain revealed that neuronal progenitor cells of the dorsal brainstem are distinctly sensitive to this pathway and serve as the cell of origin for Wnt group medulloblastomas [7]. Analogous studies of molecular aberrations distinct to non-Shh/non-Wnt tumors are conspicuously absent, as the focal molecular events specific to these tumors have only recently been described [4], [5].

*OTX2* (*Orthodenticle Homeobox 2*) is the most frequent target of focal gain in the medulloblastoma genome [3], [9], and this event occurs almost exclusively in non-Shh/non-Wnt medulloblastomas [4], [9]. *OTX2* encodes a homeobox transcription factor best known for its role in developmental patterning [10]. Its expression is dynamically regulated in the embryo [11] and restricted to the retinal pigment epithelium, pineal gland, and choroid plexus in the adult [11]. Of transformed tissues, Otx2 is expressed only in medulloblastoma and retinoblastoma [12], attesting to its lineage specificity. In established medulloblastomas, Otx2 inhibits differentiation [13] and can contribute to tumor progression [9]. Proposed mechanisms for the oncogenic function of Otx2 include transactivation of cell cycle genes [13] and induction of the *MYC* oncogene [9], which seems to play a key role in tumor maintenance in some medulloblastomas. The role of Otx2 in the initiation of medulloblastoma is less clear; however, its uniform expression pattern among non-Shh tumors [9] suggests that Otx2 expression may be acquired during clonal expansion of a preneoplastic lesion. Additionally, dynamic expression of Otx2 in cell populations destined to contribute to the mature hindbrain [11], [14] indicates a potential for the dysregulation of this gene to contribute to tumorigenesis from an Otx2-expressing cell of origin.

Studies of Otx2 in the developing mouse brain have revealed highly context-dependent functions of this gene in normal central nervous system (CNS) tissues. Otx2 plays a critical role in positioning the isthmic organizer located just rostral to the presumptive cerebellum [10], whereas truncation or extension of the Otx2 expression domain during midbrain/hindbrain specification can shift or delete cerebellar structures, respectively [15], [16]. Later in gestation Otx2 maintains compartmentalization of the neural tube by contributing to the transcription factor “code" that serves to maintain regional identity [17]. Otx2 maintains the proliferative capacity of progenitor cells in the ventral mesencephalon, which can be enhanced or suppressed by alteration of Otx2 dosage [17]. In the thalamus, Otx2 appears to suppress growth [18], underscoring the contribution of tissue context in modulating Otx2 function. In the present study, we have sought to determine the consequences of ectopic Otx2 expression in the mouse hindbrain to identify the cell types and developmental processes affected by dysregulated Otx2 as is observed in medulloblastoma.

## Methods

### Ethics Statement

This study was carried out in accordance with the recommendations from the Duke University Institutional Animal Care and Use Committee (DU IACUC). The protocols were approved by DU IACUC (Protocols A109-10-04 and A111-08-04).

### Animal care

Animals were maintained in standard conditions. *hGFAP-Cre* [*19*] and *MATH1-GFP* [*20*] transgenic animals were kindly provided by Rob Wechsler-Reya at Duke University. All animals were of mixed strain composition including strains 129 Sv/Ev, C57Bl6/J, FVB/N, and DBA/2. Genotyping primers are available upon request.

### Generation of lox-stop-lox-Otx2 knock-in mice

cDNA encoding wild type mouse Otx2 was first inserted downstream of the loxP-neo-4xpolyA-loxP fragment of the pBigT construct [21]. The resulting PacI/AscI fragment was then inserted into a modified ROSA26 acceptor plasmid containing an SfiI linearization site. Finally, the chicken actin gene (CAG) promoter was inserted into the PacI site immediately upstream of the loxP-neo-4xpolyA-loxP fragment. The resulting construct (Fig. S1A) was linearized with SfiI and electroporated into 129 Sv/Ev ES cells. G418-resistant colonies were screened by PCR, and positive colonies (18/384) were expanded for subsequent Southern blotting. Finally, expanded positive colonies were electroporated with an expression plasmid encoding NLS-Cre (NLS, nuclear localization signal fusion), and two days later RNA was purified from cultures for confirmation of OTX2 mRNA induction. Positive ES cells were injected into C57Bl6 blastocysts, and resulting chimeras were bred with C57Bl6 mice. Positive heterozygous offspring were identified by PCR from tail snip DNA.

### Real-Time PCR

Total RNA from mouse ES cells or whole cerebella was purified using the Qiagen RNAeasy kit (Hilden, Germany). Reverse transcription was carried out on 1 µg of RNA using the Bio-Rad iScript cDNA synthesis kit (Bio-Rad, Hercules, CA). Amplification and SYBR green detection was performed using an Applied Biosystems 7900HT Fast Real-Time PCR System (ABI, Carlsbad, CA) and Kapa reagents (Kapa Biosystems, Cape Town, South Africa). Real time PCR primers are available upon request.

### Tissue preparation and staining

Mice were transcardially perfused (P7 and later timepoints) first with PBS and then with 10% neutral buffered formalin (NBF). Whole brains were removed and then fixed in 10% NBF overnight (18–24 h). For quantitative studies, tissues were then paraffin embedded using standard techniques and sectioned sagittally or coronally every 300 µm to ensure sampling throughout cerebella or ventricular systems, respectively. Hematoxylin and eosin (H & E)-stained sections were examined for presence of ectopia, defined as clusters of basophilic cells in hindbrain white matter that are not found in any sections of wild type mice. For immunohistochemistry, NBF-fixed brains were cryoprotected in 20% sucrose in PBS, embedded in Optimal Cutting Temperature media, and then cut into 14 µm sections using a Shandon SME Cryotome Cryostat (Thermo Shandon, Pittsburgh, PA). Immunohistochemistry was performed using standard techniques. Slides were imaged using a Leica DMD108 imaging device (Leica, Wetzlar, Germany) for light microscopy and a Nikon Eclipse TE2000-E inverted microscope (Nikon, Tokyo, Japan) running Metamorph version 6.2r6 for fluorescent images. All fluorescent images were processed identically for brightness using Adobe Photoshop Elements (Adobe, San Jose, CA). The following antibodies were used for immunohistochemistry: goat anti-Otx2 (5 µg/mL) and Phycoerythrin-labelled mouse anti-O4 (1∶5) from R & D Systems (Minneapolis, MN), mouse anti-Ki67 (1∶100) and mouse anti-Ctnnb1 (1∶250) from BD (Franklin Lakes, NJ), mouse anti-NeuN (1∶100 on paraffin-embedded sections) and rabbit anti-Blbp (1∶500) from Millipore (Billerica, MA), rabbit anti-Pax6 (1∶300) from Covance (Princeton, NJ), mouse anti-S100β (1∶1000) from Sigma (St. Louis, MO), rabbit anti-Pax2 (1∶500) from Invitrogen (Carlsbad, CA), rabbit anti-Zic1 (1∶400) from Rockland (Gilbertsville, PA), and rabbit anti-cleaved Caspase 3 (1∶100) from Abcam (Cambridge, UK). Specificity of antibody staining was confirmed by comparing the staining of non-immune immunoglobulins at equivalent concentrations in adjacent sections.

## Results

### Generation and validation of mice constitutively expressing Otx2 in the hindbrain

To study the effect of Otx2 overexpression in the hindbrain, we generated a strain of mice in which Otx2 expression could be induced by Cre recombinase by targeting a lox-stop-lox-OTX2 construct to the ROSA26 locus of the mouse genome (Fig. S1A–C, hereafter referred to as ROSA26 *^Lsl-OTX2^*). As the cell of origin for non-Shh/non-Wnt tumors is not known, we sought to induce Otx2 in various cell types of the cerebellum and brainstem. As such, we generated *hGFAP-cre*, *ROSA26 ^Lsl-OTX2/Lsl-OTX2^* animals, hereafter referred to as GFAP:Hi-Otx2 mice, to induce Otx2 expression throughout the brain following the establishment of the hindbrain primordium [8], [19]. Enhancement of Otx2 expression in whole cerebellum lysate was validated by qPCR (Fig. S1D) and Western Blotting (not shown).

**Figure 1:**
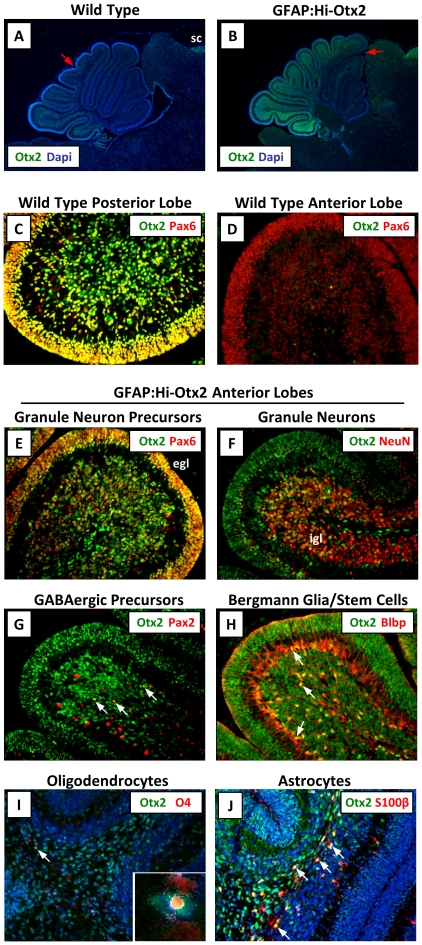
Otx2 expression is induced in various cell types in GFAP:Hi-Otx2 mice. Sections from P7 wild type (A, C–D) or GFAP:Hi-Otx2 (B, E–J) mice were immunostained with the indicated antibodies. For (A) and (B), anterior expression threshold is designated with a red arrow. (A, B) are shown at 4× magnification (mag), all others 20× mag. sc, superior colliculus; egl, external granule layer; igl, internal granule layer. White arrows indicate overlapping expression of the indicated markers in individual cells.

Immunohistochemistry was performed on P7 brains to verify that Otx2 was induced in expected cell types and tissue domains. We noted that Otx2 expression in hindbrains of *ROSA26 ^Lsl-OTX2/Lsl-OTX2^* mice (e.g. lacking the Cre transgene, hereafter referred to as “wild type mice") was mostly limited to GNPs and mature granule neurons of the posterior lobes of the cerebellum (Fig. 1A & C–D, Fig. S2A–E), in agreement with previous *in situ* hybridization studies in the rat [14]. In GFAP:Hi-Otx2 mice, this expression domain was extended into more anterior lobes of the cerebellum and into the white matter of the cerebellum and brainstem (Fig. 1B) and was expressed in Pax6^+^ GNPs, NeuN^+^ differentiated granule neurons, Pax2^+^ GABAergic neuron precursors, S100β^+^ astrocytes, O4^+^ oligodendrocytes, and Blbp^+^ Bergmann glia/stem cells (Fig. 1E–J).

### Enforced Otx2 expression gives rise to proliferative hyperplasias in the cerebellum and brainstem

GFAP:Hi-Otx2 mice exhibited decreased postnatal growth relative to wild type mice (data not shown), and female GFAP:Hi-Otx2 animals were hypofertile. There was a trend towards reduced overall survival (Fig. S3; *p* = 0.11, log-rank test), whereas moribund animals exhibited lethargy but no overt CNS symptoms such as head doming or ataxia. There were no histological indications of brain tumors in moribund or asymptomatic GFAP:Hi-Otx2 mice aged for up to one year. An analysis of lateral ventricle size of P21 mice revealed expansion of ventricular width of 150% or more relative to wild type in 3/4 GFAP:Hi-Otx2 animals (data not shown), leading us to conclude that hydrocephalus is likely the cause of shortened lifespan in these mice.

**Figure 2:**
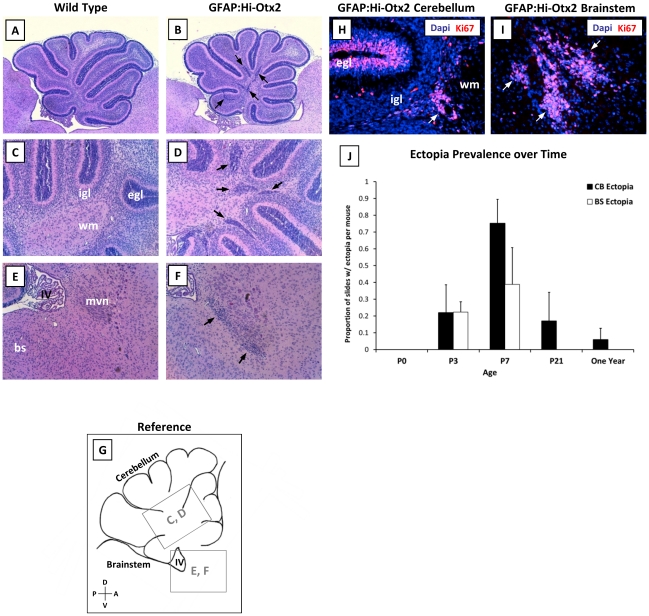
GFAP:Hi-Otx2 mice develop focal hyperplasias in the cerebellar white matter and the brainstem. (A–F) H & E stained sections from P7 hindbrains of (A, C, E) wild type and (B, D, F) GFAP:Hi-Otx2 mice. Fields shown are as follows: (A, B) whole cerebella at 4× magnification (mag), (C, D) cerebellar white matter at 10× mag, (E, F) dorsal brainstem at 10× mag. (G) reference illustration of fields shown in C–F indicated by grey boxes. (H, I) Immunofluorescent staining for Ki67 in (H) cerebellar ectopia and (I) brainstem ectopia in GFAP:Hi-Otx2 mice (20× mag). (J) Prevalence of ectopia in GFAP:Hi-Otx2 mice over time. CB, cerebellum; BS/bs, brainstem; egl, external granule layer; igl, internal granule layer; wm, white matter; IV, fourth ventricle; mvn, medial vestibular nuclei; D, dorsal; V, ventral; A, anterior; P, posterior. Black or white arrows indicate ectopia.

To identify developmental aberrations resulting from Otx2 expression, brains of GFAP:Hi-Otx2 mice and wild type littermates were harvested at various timepoints from birth until the completion of hindbrain development (P0, P3, P7, P21). Hindbrains of GFAP:Hi-Otx2 mice were indistinguishable from those of wild type mice at P0; however, at P3 and P7, focal hyperplasias had accumulated in the cerebellar white matter (hereafter referred to as “cerebellar ectopia", Fig. 2A–D) and at the dorsal aspect of the pons near the floor of the IVth ventricle (“brainstem ectopia" hereafter; Fig. 2E–G) in GFAP:Hi-Otx2 mice. Ki67 staining of ectopia in P7 mice revealed that these hyperplasias were proliferative (Fig. 2H–I). All GFAP:Hi-Otx2 mice developed cerebellar ectopia (*n* = 8), which were present in nearly every sagittal section by P7 (Fig. 2J) and reached a peak density of 2.5±1.2 ectopia/slide/mouse. In contrast, brainstem ectopia were identified in 7/8 mice and accumulated at more limited coordinates just lateral to the midline (data not shown).

### Otx2 distinctly affects neuronal progenitor cells

**Figure 3:**
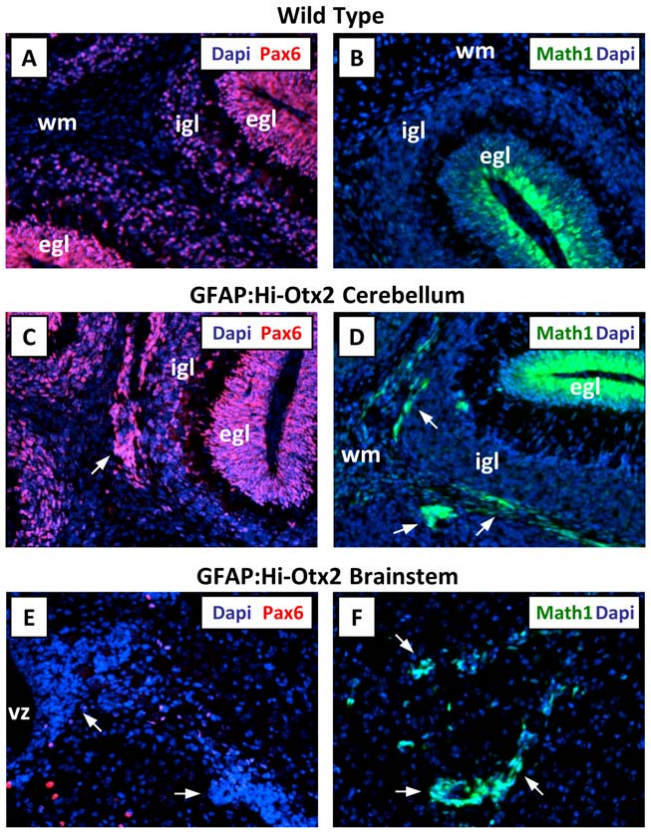
Cerebellar and brainstem ectopia express Math1. (A, C, E) Immunofluorescent staining for Pax6 in (A) wild type cerebellum, (C) GFAP:Hi-Otx2 cerebellum, and (E) GFAP:Hi-Otx2 brainstem. (B) MATH1-GFP reporter expression in the cerebellum of *MATH1-GFP* transgenic mice. (D, F) MATH1-GFP reporter expression in the (D) cerebellum and (F) brainstem of GFAP:Hi-Otx2, *MATH1-GFP* mice. 20× mag. Arrows indicate ectopia.

Because Otx2 was expressed broadly in GFAP:Hi-Otx2 mice, determining the cell types comprising these ectopia would identify the cell types affected by Otx2 overexpression. To this end, we stained sections from GFAP:Hi-Otx2 mice with various lineage markers and examined MATH1-GFP reporter expression in GFAP:Hi-Otx2, *MATH1-GFP* transgenic mice [20]. *MATH1-GFP* mice harbor a GFP reporter allele driven by the *MATH1* promoter, which identifies rhombic lip-derived glutamatergic neuronal progenitor cells including cerebellar GNPs and neuronal progenitors of the precerebellar system. Cerebellar ectopia stained for the GNP marker Pax6 (Fig. 3A & C) and expressed the MATH1-GFP reporter allele (Fig. 3B, D) but did not stain for markers of postmitotic glutamatergic neurons (Zic1), GABAergic neuron precursors (Pax2), astrocytes (S100β), oligodendrocytes (O4), or Bergmann glia/postnatal stem cells (Blbp, Fig. S4A–E). Brainstem ectopia did not stain for any of the lineage markers tested (Fig. 3E and Fig. S4G–K) but did express the MATH1-GFP reporter allele (Fig. 3F). These findings indicate that ectopia of both compartments resemble neuronal progenitor cells, whereas cerebellar ectopia specifically resemble GNPs. Examination of the cellular organization and morphology of each cell type identified with immunohistochemistry did not reveal consistent aberrations of other cell types in the cerebellum or brainstem in GFAP:Hi-Otx2 mice (Fig. 1 and data not shown). As expected, all ectopia stained for Otx2 protein (Fig. S4F, L).

**Figure 4:**
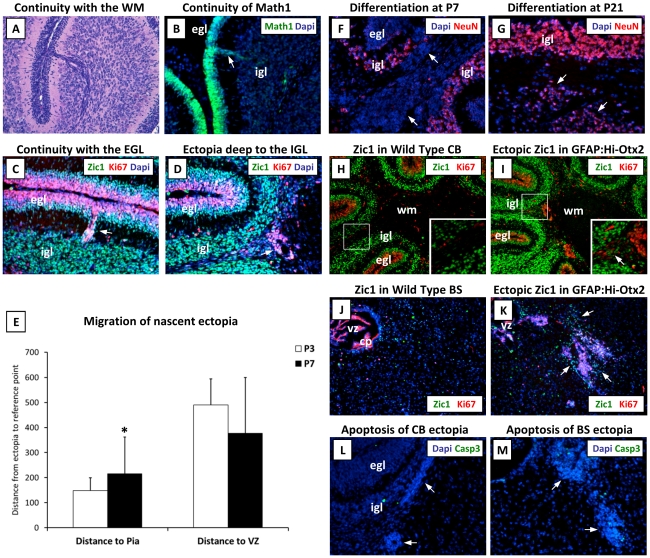
Origin and fate of cerebellar ectopia. (A) Rare ectopia continuous with the EGL, H & E staining. (B) Image of rare Math1^+^ ectopia continuous with the EGL in GFAP:Hi-Otx2, *MATH1-GFP* mice. (C–D, F–M) Images of GFAP:Hi-Otx2 brain sections stained with the indicated antibodies. (C–D) Sections immunostained with Ki67 and Zic1 of (C) rare ectopia continuous with the EGL and (D) representative ectopia located just deep to the IGL. (E) Localization of histologically-apparent ectopia of GFAP:Hi-Otx2 mice at P3 and P7 (distance in µm). (F, G) Expression of differentiation markers in cerebellar ectopia at (F) P7 and (G) P21. (H–K) Location of partially- to fully-differentiated neurons in (H, I) cerebellum or (J, K) brainstem as evidenced by Zic1 and Ki67 staining shown at 10× mag in (H, J) wild type mice or (I, K) GFAP:Hi-Otx2 mice. (L, M) Cleaved caspase-3 staining of ectopia in the (L) cerebellum and (M) brainstem. All images except (H–K) shown at 20× mag. CB, cerebellum; BS, brainstem. Asterisk indicates *p*≤0.05 relative to P3, student's t-test. Arrows indicate ectopia. Boxes indicate location of cropped and zoomed image.

We then sought to determine the spatial origin of ectopic cerebellar GNPs, which could have been derived from the EGL, the rhombic lip, or from stem cells in the white matter [22]. Histological examination of sections from P7 GFAP:Hi-Otx2 mice revealed rare streams of basophilic cells continuous with the EGL and either the IGL or white matter (Fig. 4A). Detection of the MATH1-GFP reporter allele and immunostaining revealed that these cells resembled proliferative GNPs continuous with the outer EGL (Fig. 4B–D). Additionally, spatial localization of ectopia at P3 revealed a closer average distance to the cerebellar surface relative to P7 ectopia (Fig. 4E). Immunohistochemistry of the ventricular zone and rhombic lip at P0 demonstrated a normal pattern of proliferation and GNP localization in GFAP:Hi-Otx2 mice at this timepoint (data not shown). Collectively, these findings indicate that cerebellar ectopia are most likely derived from inward migration of GNPs from the EGL.

### Ectopia are resolved through differentiation

To determine the fate of ectopia, we next immunostained GFAP:Hi-Otx2 sections for markers of apoptosis or neuronal differentiation. Examination of cerebellar ectopia at P21 revealed a lower proportion of Ki67^+^ cells (data not shown) and induction of the postmitotic marker NeuN (Fig. 4F–G), suggesting that ectopia are eventually resolved through differentiation. This is supported by the observation that ectopic Zic1^+^ cells representing partially-differentiated neurons were often found at the periphery of ectopia in the white matter of both the cerebellum and brainstem at P7 (Fig. 4H–K), indicative of continuous differentiation and dispersal of cells away from these hyperplasias. Ectopia at either site did not exhibit frequent Caspase-3 cleavage (Fig. 4L–M) or histological indicators of necrosis at P21 and earlier timepoints (data not shown), further supporting this interpretation.

### Otx2-induced hyperplasia is spatially distinct from Shh pathway-induced hyperplasia

**Figure 5:**
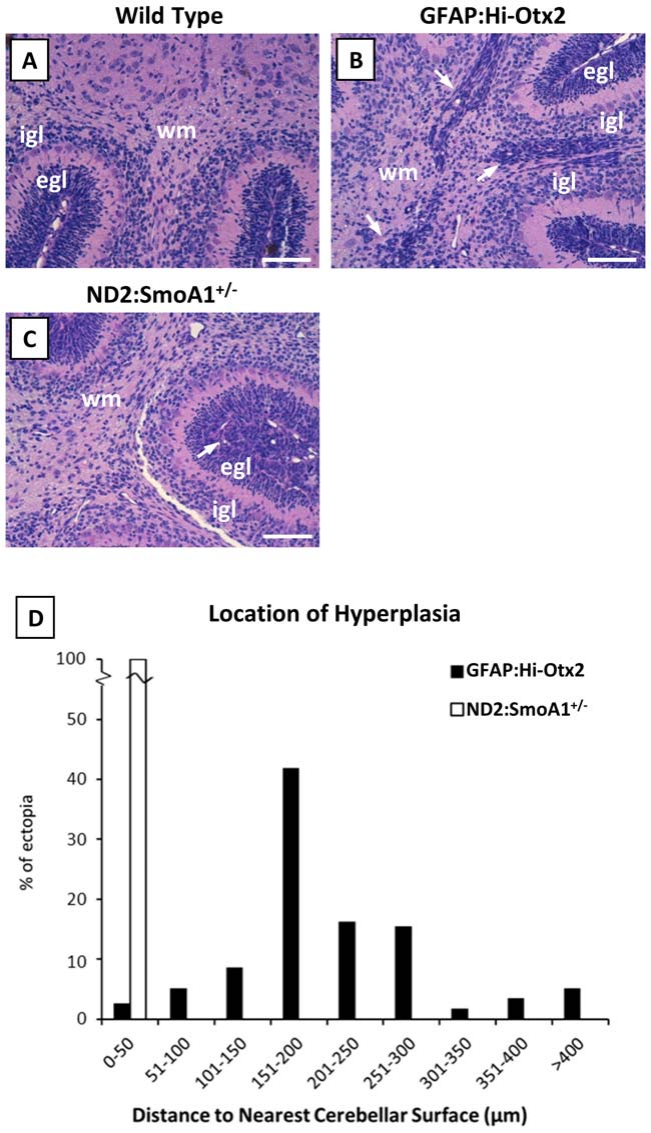
Cerebellar hyperplasias in GFAP:Hi-Otx2 mice are spatially distinct from dysplasia/hyperplasia observed in *ND2:SmoA1* ^+/−^ mice. Images of H & E stained sections (20× mag) from (A) wild type mice, (B) GFAP:Hi-Otx2 mice, and (C) *ND2:SmoA1^+/−^* mice. Scale bar: 100 µm. (D) Location of ectopia at P7 in GFAP:Hi-Otx2 mice relative to location of hyperplasia/dysplasia in P7 ND2:SmoA1^+/−^ mice. Arrows indicate regions of hyperplasia.

Otx2-induced ectopia of the cerebellum and brainstem were reminiscent of preneoplastic cells identified in animal models of Shh and Wnt group medulloblastomas, respectively. In order to compare cerebellar ectopia with preneoplastic cells of Shh group medulloblastomas, we harvested brains from P7 *ND2:SmoA1* ^+/−^ mice, which express oncogenic Smoothened in GNPs [23]. We noted dysplasia at the outer EGL and hyperplasias extending into the inner EGL in these mice, but we did not observe invasion of GNPs deep into the cerebellum (Fig. 5A, C–D). In contrast, hyperplasias of GFAP:Hi-Otx2 mice were predominately located deep in the cerebellum at the periphery of the white matter (Fig. 5B–D).

## Discussion

In this study, we have determined that the medulloblastoma oncogene Otx2 promotes enhanced migration and persistent proliferation of hindbrain neuronal progenitor cells away from their mitogenic niches. This selective growth advantage conferred upon normal hindbrain cell types by Otx2 reveals the potential for this gene to contribute to early stages of medulloblastoma tumorigenesis by altering the spatiotemporal dynamics of neuronal progenitor cell proliferation. Despite broad overexpression of Otx2, this oncogene distinctly affected cerebellar GNPs and brainstem neuronal progenitors, both of which are known cell origins of medulloblastoma [7], [8].

Precise regulation of GNP proliferation, migration, and differentiation is critical to both cerebellar function and medulloblastoma tumor suppression [6]. The mitogenic niche for GNPs, the outer EGL, gives rise to postmitotic GNPs of the inner EGL, which then migrate inward to form a layer of differentiated granule neurons comprising the IGL [24]. Mutations deregulating Shh signaling or inhibiting GNP chemotaxis result in sustained GNP proliferation at the EGL; however, cells that have left this niche exit the cell cycle and differentiate [8], [25]. Ectopic proliferation away from the EGL has been previously described for GNPs lacking *RB* [*26*], implicating the G1/S cell cycle checkpoint in restricting GNP proliferation to the EGL. Otx2 has been shown to promote cell cycle progression and inhibit differentiation in medulloblastoma cells [13], thus it is possible that cell cycle deregulation by Otx2 underlies both tumor maintenance in established medulloblastomas [9], [13] and mitogenic niche-independent proliferation of neuronal progenitor cells in GFAP:Hi-Otx2 mice. Ectopic GNP migration into the cerebellar white matter of animals deficient for the SDF-1 receptor *CXCR4* [27] or the Netrin receptor *UNC5C* [28], [29] demonstrate a role for chemokines in directing GNP migration. Otx2 has not been previously implicated in coordinating either cell migration or chemokine signaling, so the mechanistic basis for the effect of Otx2 upon migration of neuronal progenitor cells is unclear. In addition to its potential to contribute to tumor initiation, a role for Otx2 in cell migration could serve as a mechanism of tumor metastasis, consistent with the known association between *OTX2* and tumor progression [9]. It should be noted that in posterior lobes of the cerebellum, endogenous Otx2 is expressed in both GNPs and differentiated granule neurons, indicating that Otx2 expression is compatible with proper spatial restriction of GNP proliferation in some locations and that cellular context is a critical mediator of Otx2 function.

The formation of precerebellar nuclei in the brainstem involves progenitor cell specification at the rhombic lip, cell cycle exit, and subsequent migration via well-defined routes [24]. Constitutive activation of the Wnt pathway in precerebellar neuronal progenitor cells inhibits migration away from their mitogenic niche, resulting in hyperplasia at the surface of the lower rhombic lip [7]. Otx2 did not activate the Wnt pathway (Fig. S5A–C), and brainstem hyperplasias induced by Otx2 were found rostral to and deeper than the lower rhombic lip. It is thus likely that Otx2 affects mechanisms spatially restricting proliferation that are common to the cerebellum and brainstem while affecting factors accounting for proper migration that are distinct to each compartment.

In GFAP:Hi-Otx2 mice, both cerebellar and brainstem ectopia differentiated as the animals reached adulthood, and thus it appears that the mechanisms limiting proliferative lifespan of neuronal progenitors are intact and serve as the major limiting factor to transformation. This is particularly apparent for cerebellar ectopia, whose duration of proliferation is in close agreement with that of GNPs in wild type animals. At their peak abundance, hyperplasias induced by Otx2 are in fact more readily detectable than those of *ND2:SmoA1* ^+/−^ mice (unpublished observation), suggesting that the limited transformation potential of Otx2 relative to other oncogenes is a consequence of the duration rather than the scale of its effect. Although Otx2-induced hyperplasias share many characteristics of medulloblastoma precursor cells, it is yet to be determined whether these ectopia are indeed preneoplastic lesions. To this end, further studies will be required to demonstrate that hindbrain neuronal progenitor cells can be transformed by a combination of Otx2 and as yet unidentified cooperating genetic events. This work thus establishes a basis for future studies characterizing the pathogenesis of non-Shh/non-Wnt medulloblastomas in relation to the phenotypes induced by Otx2 described herein.

## Supporting Information

Figure S1 Generation of Lox-stop-lox-Otx2 knockin mice. (A) ROSA26 locus (top) and pROSA26-CAG-lox-stop-lox-OTX2 targeting construct (below). “Probe" indicates location of probe for Southern blot. (B) Southern blot of EcoRV-digested genomic DNA from three representative ROSA26 *^Lsl-OTX2/+^* ES cell clones (3A3, 3A7, 3B9) and wild type ES cells. (C) OTX2 mRNA expression levels as determined by qPCR in wild type and ROSA26 *^Lsl-OTX2/+^* clones (3A3 and 3B9) electroporated with a Cre expression plasmid. (D) OTX2 mRNA expression level in whole cerebella from adult wild type and GFAP:Hi-Otx2 mice. WT, wild type. UT, untreated. Asterisk indicates *p*≤0.05 relative to (C) untreated or (D) wild type.
(PDF)

Figure S2 Endogenous Otx2 expression is mostly restricted to GNPs and mature granule neurons. (A–E) Sections from P7 wild type mice were immunostained with the indicated antibodies, 20× magnification (mag) of posterior lobes are shown. egl, external granule layer. igl, internal granule layer. Arrows indicate overlapping expression of the indicated markers in individual cells.
(PDF)

Figure S3 Survival of GFAP:Hi-Otx2 mice. Animals were monitored from birth and sacrificed when moribund. *p* = 0.11, Log-rank test.
(PDF)

Figure S4 Cerebellum and brainstem ectopia do not stain for lineage markers other than Math1/Pax6. Immunofluorescent images of ectopia from the cerebellum (A–F) and brainstem (G–L) of P7 GFAP:Hi-Otx2 mice stained with the indicated cell lineage markers or for Otx2 protein. 20× magnification (mag).
(PDF)

Figure S5 Otx2 does not induce Wnt pathway activation. Immunofluorescent staining (20× mag) for Ctnnb1 in ectopia of (A) the brainstem or (B) cerebellar white matter in GFAP:Hi-Otx2 mice. (C) Positive antibody control staining of mouse colon.
(PDF)
