# Supplementary material for: Aberrant Otx2 Expression Enhances Migration and Induces Ectopic Proliferation of Hindbrain Neuronal Progenitor Cells
Source: PLoS One. 2012 Apr 27;7(4):e36211. doi: 10.1371/journal.pone.0036211 (PMC3338642; doi:10.1371/journal.pone.0036211)
Supplement: Figure S1 — Generation of Lox-stop-lox-Otx2 knockin mice. (A) ROSA26 locus (top) and pROSA26-CAG-lox-stop-lox-OTX2 targeting construct (below). “Probe" indicates location of probe for Southern blot. (B) Southern blot of EcoRV-digested genomic DNA from three representative ROSA26 Lsl-OTX2/+ ES cell clones (3A3, 3A7, 3B9) and wild type ES cells. (C) OTX2 mRNA expression levels as determined by qPCR in wild type and ROSA26 Lsl-OTX2/+ clones (3A3 and 3B9) electroporated with a Cre expression plasmid. (D) OTX2 mRNA expression level in whole cerebella from adult wild type and GFAP:Hi-Otx2 mice. WT, wild type. UT, untreated. Asterisk indicates p≤0.05 relative to (C) untreated or (D) wild type. (PDF) [file pone.0036211.s001.pdf]

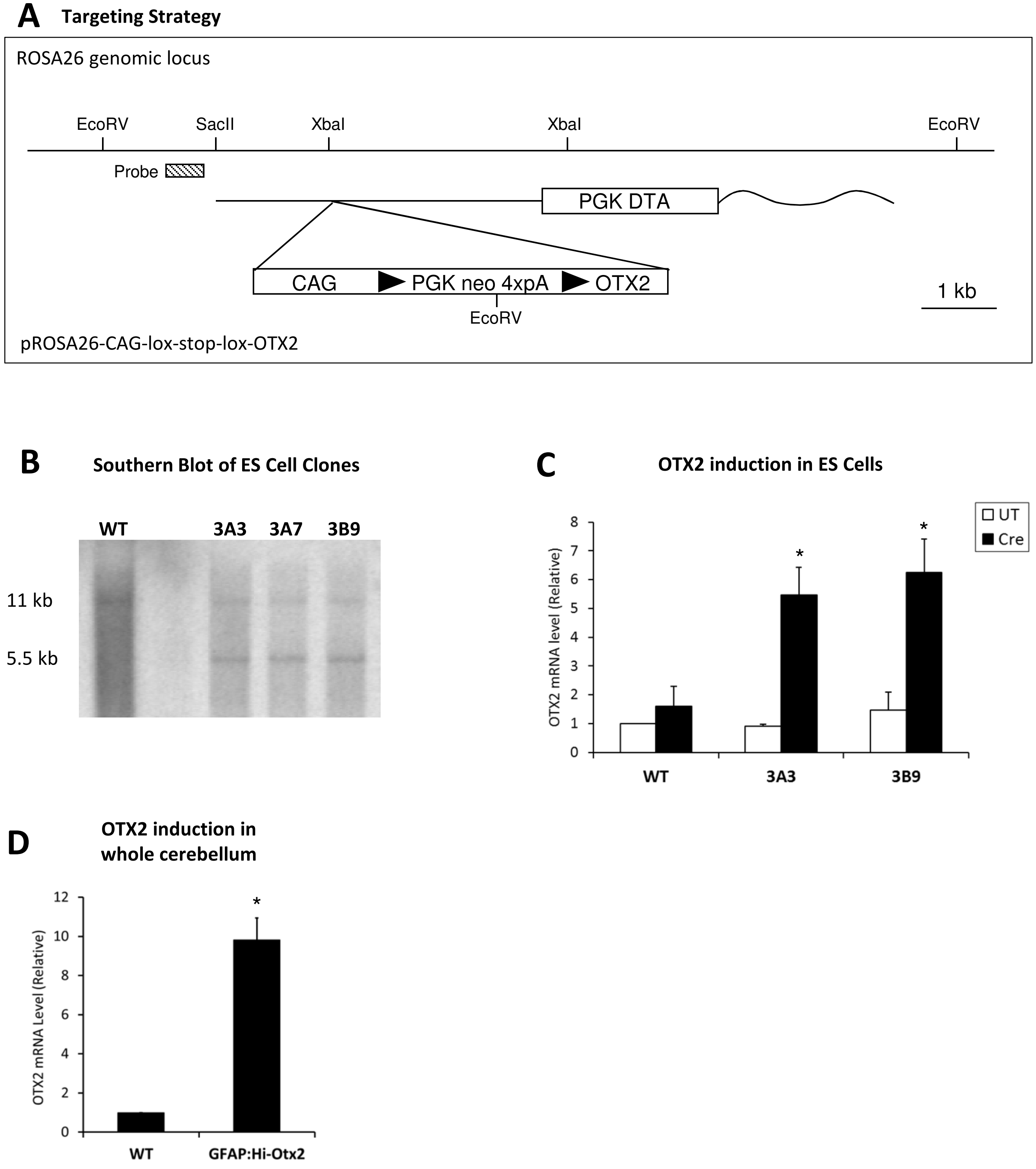
A
Targeting Strategy
ROSA26 genomic locus
EcoRV
SacII
XbaI
XbaI
EcoRV
Probe
PGK DTA
CAG
PGK neo 4xpA
OTX2
1 kb
EcoRV
pROSA26-CAG-lox-stop-lox-OTX2
B
Southern Blot of ES Cell Clones
WT
3A3
3A7
3B9
11 kb
5.5 kb
C
OTX2 induction in ES Cells
UT
Cre
OTX2 mRNA level (Relative)
0
1
2
3
4
5
6
7
8
*
*
WT
3A3
3B9
D
OTX2 induction in whole cerebellum
OTX2 mRNA Level (Relative)
0
2
4
6
8
10
12
*
WT
GFAP:Hi-Otx2
