# Supplementary material for: Aberrant Otx2 Expression Enhances Migration and Induces Ectopic Proliferation of Hindbrain Neuronal Progenitor Cells
Source: PLoS One. 2012 Apr 27;7(4):e36211. doi: 10.1371/journal.pone.0036211 (PMC3338642; doi:10.1371/journal.pone.0036211)
Supplement: Figure S2 — Endogenous Otx2 expression is mostly restricted to GNPs and mature granule neurons. (A–E) Sections from P7 wild type mice were immunostained with the indicated antibodies, 20× magnification (mag) of posterior lobes are shown. egl, external granule layer. igl, internal granule layer. Arrows indicate overlapping expression of the indicated markers in individual cells. (PDF) [file pone.0036211.s002.pdf]

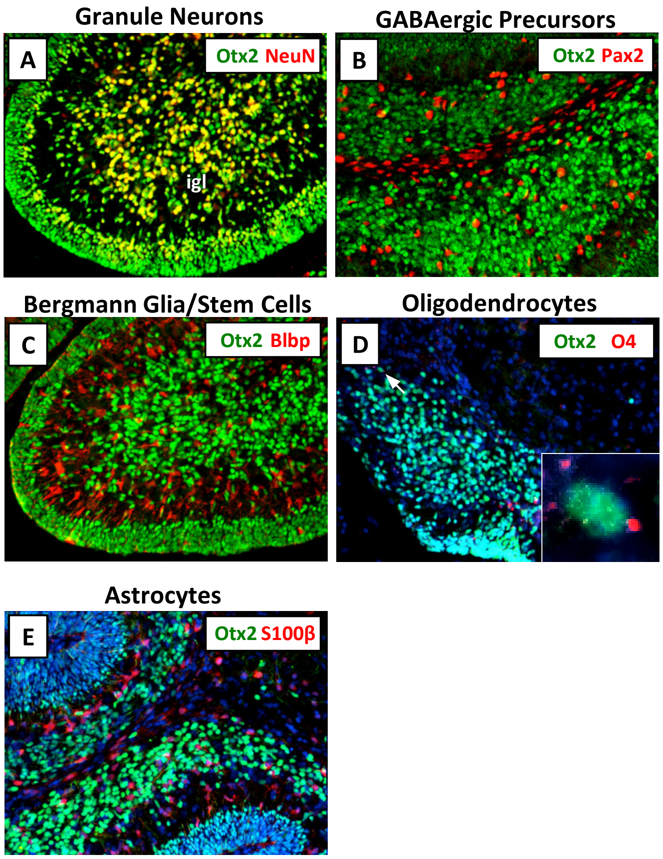
Granule Neurons
A
Otx2 NeuN
igl
GABAergic Precursors
B
Otx2 Pax2
Bergmann Glia/Stem Cells
C
Otx2 Blbp
Oligodendrocytes
D
Otx2 O4
Astrocytes
E
Otx2 S100β
