# Supplementary figures and images for: Aberrant Otx2 Expression Enhances Migration and Induces Ectopic Proliferation of Hindbrain Neuronal Progenitor Cells
Source: PLoS One. 2012 Apr 27;7(4):e36211. doi: 10.1371/journal.pone.0036211 (PMC3338642; doi:10.1371/journal.pone.0036211)

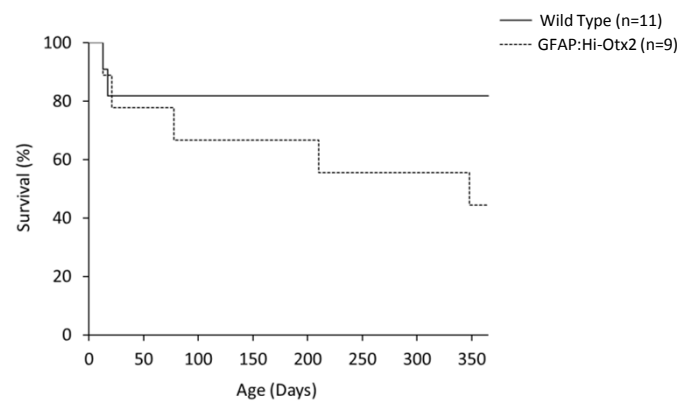

Supplement: Figure S3 — Survival of GFAP:Hi-Otx2 mice. Animals were monitored from birth and sacrificed when moribund. p = 0.11, Log-rank test. (PDF) [file pone.0036211.s003.pdf]

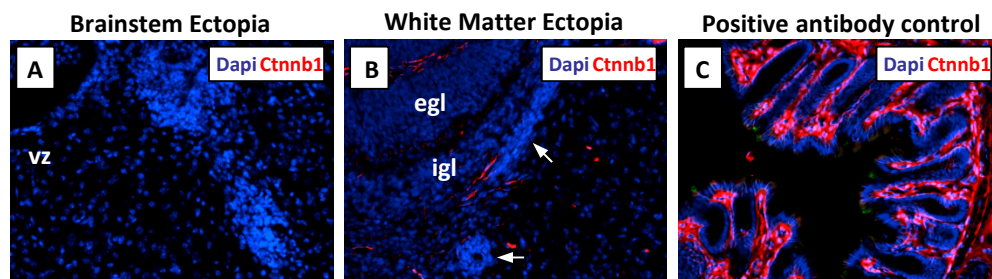

Supplement: Figure S5 — Otx2 does not induce Wnt pathway activation. Immunofluorescent staining (20× mag) for Ctnnb1 in ectopia of (A) the brainstem or (B) cerebellar white matter in GFAP:Hi-Otx2 mice. (C) Positive antibody control staining of mouse colon. (PDF) [file pone.0036211.s005.pdf]
