# Supplementary material for: Aberrant Otx2 Expression Enhances Migration and Induces Ectopic Proliferation of Hindbrain Neuronal Progenitor Cells
Source: PLoS One. 2012 Apr 27;7(4):e36211. doi: 10.1371/journal.pone.0036211 (PMC3338642; doi:10.1371/journal.pone.0036211)
Supplement: Figure S4 — Cerebellum and brainstem ectopia do not stain for lineage markers other than Math1/Pax6. Immunofluorescent images of ectopia from the cerebellum (A–F) and brainstem (G–L) of P7 GFAP:Hi-Otx2 mice stained with the indicated cell lineage markers or for Otx2 protein. 20× magnification (mag). (PDF) [file pone.0036211.s004.pdf]

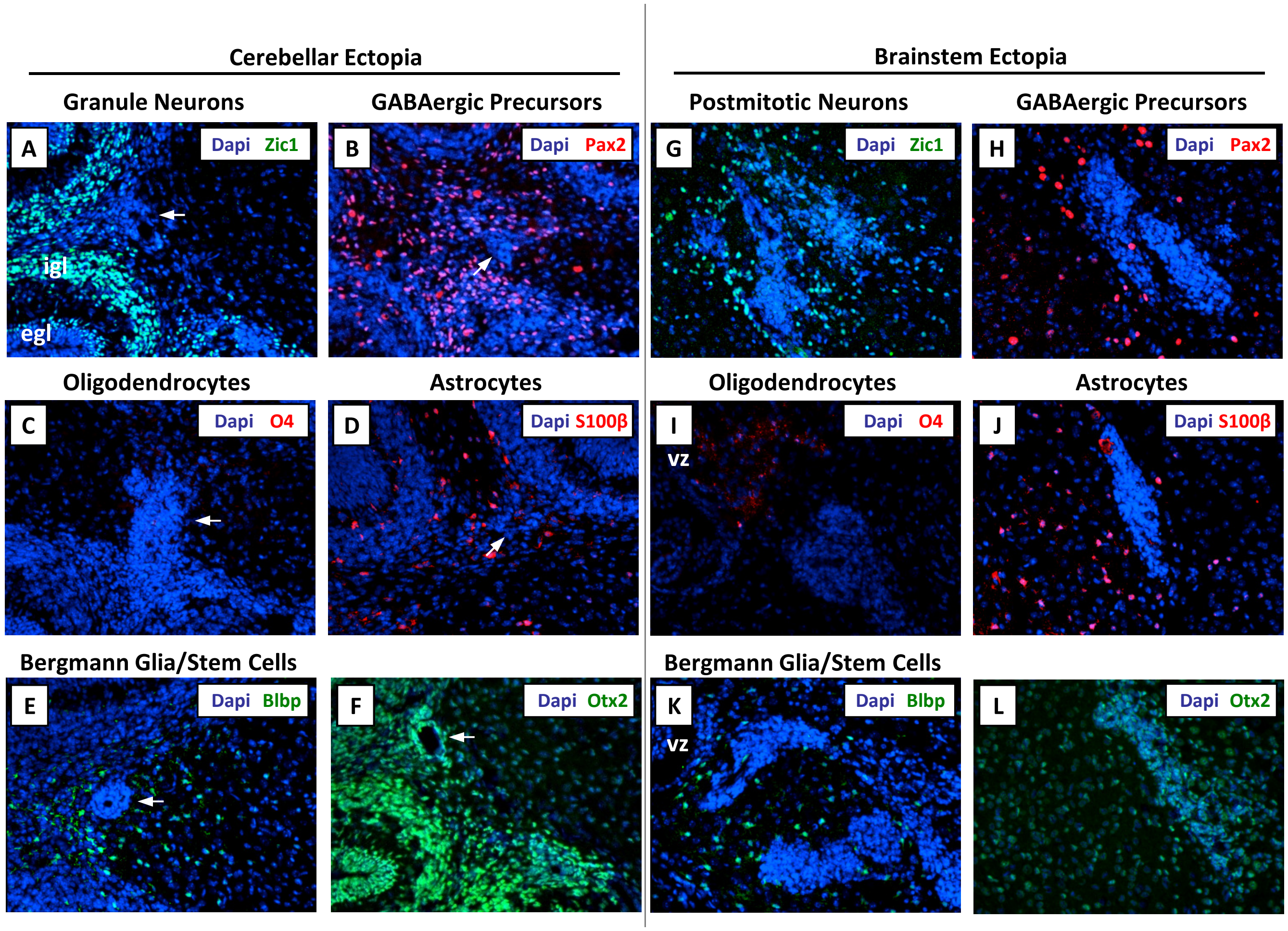

Cerebellar Ectopia
Brainstem Ectopia
Granule Neurons
GABAergic Precursors
Postmitotic Neurons
GABAergic Precursors
A
Dapi Zic1
igl
egl
B
Dapi Pax2
G
Dapi Zic1
H
Dapi Pax2
Oligodendrocytes
Astrocytes
Oligodendrocytes
Astrocytes
C
Dapi O4
D
Dapi S100β
I
Dapi O4
VZ
J
Dapi S100β
Bergmann Glia/Stem Cells
Bergmann Glia/Stem Cells
E
Dapi Blbp
F
Dapi Otx2
K
Dapi Blbp
VZ
L
Dapi Otx2
